# Advanced Robotics to Address the Translational Gap in Tendon Engineering

**DOI:** 10.34133/2022/9842169

**Published:** 2022-09-15

**Authors:** Iain L. Sander, Nicole Dvorak, Julie A. Stebbins, Andrew J. Carr, Pierre-Alexis Mouthuy

**Affiliations:** ^1^Botnar Research Centre, Nuffield Department of Orthopaedics, Rheumatology, and Musculoskeletal Sciences, University of Oxford, Windmill Road, Oxford OX3 7LD, UK; ^2^Oxford Gait Laboratory, Nuffield Orthopaedic Centre, Tebbit Centre, Windmill Road, Oxford OX3 7HE, UK

## Abstract

Tendon disease is a significant and growing burden to healthcare systems. One strategy to address this challenge is tissue engineering. A widely held view in this field is that mechanical stimulation provided to constructs should replicate the mechanical environment of native tissue as closely as possible. We review recent tendon tissue engineering studies in this article and highlight limitations of conventional uniaxial tensile bioreactors used in current literature. Advanced robotic platforms such as musculoskeletal humanoid robots and soft robotic actuators are promising technologies which may help address translational gaps in tendon tissue engineering. We suggest the proposed benefits of these technologies and identify recent studies which have worked to implement these technologies in tissue engineering. Lastly, key challenges to address in adapting these robotic technologies and proposed future research directions for tendon tissue engineering are discussed.

## 1. Introduction

Tendons are the viscoelastic connective tissues which attach the muscle to the bone (Box 1). Tendon injuries represent the highest proportion of musculoskeletal conditions for which patients seek medical treatment [1]. These injuries are painful and present a significant socioeconomic burden. The lifetime cost of a rotator cuff tear to society has been estimated at 20,000-30,000 GBP/patient in the United Kingdom and 20,000-100,000 USD/patient in the United States, with the US rotator cuff tendon repair market being estimated at 1.5 billion USD/year [2–4]. The main treatment for a full thickness rotator cuff tears is surgical repair. Although more than 250,000 rotator cuff tendon surgeries are performed per year in the United States alone, patient outcomes remain poor, with upwards of 40% of tendon repairs failing postoperatively [5]. Revision surgeries further compound the social and economic impacts of tendon disease. With the increasing prevalence of tendon injury due to an ageing population and poor treatment outcomes, there is a growing need for knowledge from other disciplines to inform and advance therapy for tendon disease.

A promising repair strategy is the development of engineered tendon grafts for clinical use. Tendon tissue engineering (TTE) involves using bioreactor systems to generate tissue *in vitro* using scaffolds, human or animal cells, and appropriate growth conditions. As demonstrated through physical therapy, providing tendon tissue with the appropriate amount and types of mechanical stimulation is essential for healthy tissue development, maintenance, and repair [6]. *In vitro* research has shown that mechanically stimulated tendon tissue demonstrates increased extracellular matrix (ECM) deposition, a greater degree of structural protein alignment, and stronger biomechanical properties when compared to statically cultured tissue [7]. A widely held opinion is that the mechanical context provided in TTE experiments should closely mirror loading experienced by native tendon tissue [7]. The lack of bioreactor systems which can recapitulate *in vivo* tendon loading (Box 2) is a major translational gap in the development of functional engineered grafts for clinical use. Despite mechanical stimulation being discussed in a number of recent TTE reviews [7–11], no clear consensus exists on the specific obstacles to providing physiologically relevant mechanical context and the technologies which may help address these obstacles.

Biomimetic robotic systems are uniquely positioned for advancing TTE. Robotic technology is already being used clinically for a variety of medical applications, including orthopaedic surgery, advanced prostheses, and powered exoskeletons for rehabilitation following neurological injury [12]. There is a growing interest in applying robotic technologies for TTE, and several recent papers have suggested convergence between these fields [13, 14]. This review article advances discussion by summarising trends in recent TTE literature, identifying current limitations to providing physiologically relevant mechanical loading, and highlighting the potential role of musculoskeletal humanoids and soft robotics in providing mechanical stimulation to engineered tendon constructs.

## 2. Conventional Tendon Tissue Engineering

Tissue engineering involves the complex interplay of gas exchange, nutrient and waste transfer, temperature and pH control, and maintenance of a sterile environment. As shown through *in vitro* studies, tendon tissue also requires appropriate mechanical context to grow and develop properly. It is important to ensure that tendon tissue constructs experience the right amount of mechanical loading; insufficient stimulation has been shown to lead to catabolic gene expression and eventual loss of structural integrity, while excess loading of tendon tissue leads to inflammation, degradative enzyme production, undesirable fibroblast differentiation, and programmed cellular death [26, 33]. *In vivo* imaging studies done in humans estimate that peak tendon strain for physiologic motion ranges from 3 to 15% depending on tendon location and individual anatomy [18, 23–25]. TTE studies prior to 2014 typically applied 2-10% strain cyclically at a frequency of 0.1-2 Hz [7]. As discussed later in this section, most bioreactors currently being used offer only uniaxial tensile loading. A list of recent *in vitro* studies using traditional bioreactor systems with human and animal cells is compiled in Table 1.

To create appropriate mechanical context for TTE constructs, it is important to have bioreactor systems that provide physiologically relevant loading, scaffolds and growth factors that induce a desirable biological response during tissue culture, and cell types that respond appropriately to mechanical stimulation. The different cell lines used in TTE are briefly discussed below as recent reviews of these subjects already exist [68–71]. This article is primarily focused on the role of bioreactor systems in developing appropriate mechanical context.

### 2.1. Cell Types Used in TTE

Cell selection in TTE is a balance of the following criteria: ability to successfully proliferate during incubation, ability to differentiate into in situ tendon cells, availability for research purposes, and no ethical or legal concerns associated with their use [68]. Although native tenocytes and *in situ* fibroblasts represent the most clinically relevant cell source for TTE, obtaining these cells can be challenging in practice. Tendon cell extraction poses a significant risk of donor morbidity and extracting a satisfactory quantity of tendon cells is challenging given their low cell number in native tissue [8]. With these practical limitations, other cell types are also currently being used for TTE research. The broad categories of cells currently being used include stem cells, tenocytes, and dermal fibroblasts [8].

Nontendon stem cells, including embryonic stem cells (ESCs), bone marrow mesenchymal stem cells (BMSCs), and adipose-derived stem cells (ADSCs), have been used in most studies listed in Table 1. Advantages of these cell types include their potential to differentiate and high proliferative capacity. Limitations for translational applicability in the use of BMSCs and ADSCs include the risk of ectopic bone formation within the constructs and senescence with increased cell passaging. Pluripotent ESCs are appealing due to their proliferative ability but have limited availability due to strict policy and ethical concerns surrounding their use. Using retroviruses or lentiviruses to generate induced pluripotent stem cells (IPSCs) is of growing interest for TTE as this technique offers unlimited proliferative ability without the ethical concerns associated with ESC tissue. Several recent studies have demonstrated tenogenic differentiation in transfected IPSCs [72, 73]. A recurrent limitation in studies using IPSCs has been the low yield of successfully transfected cells [68, 72, 73]. Song et al.'s recent study has demonstrated that biophysical cues, such as nuclear deformation, can cause epigenetic changes and eventually alter gene expression, resulting in increased yield of transfected IPSCs from a cell population [30]. As further research on this topic develops, there may be increased use of these cells in TTE.

In contrast to immature cell lines, dermal fibroblasts—used in a number of the studies listed in Table 1—are an alternative cell population commonly used in TTE [36, 51, 63]. These mature collagen producing cells have similar morphology to tenocytes and readily proliferate. These cells can be readily sourced via skin punch biopsy and have low risk of donor site morbidity [68]. Disadvantages of this cell type include the risk of fibrotic scar formation and suppression of tendon-like healing in absence of tenocyte biomarkers [7, 68]. Given these disadvantages, it is likely that these cell populations are more useful for demonstrating feasibility of response to mechanical stimulation in research settings rather than translation for eventual clinical use. There is ongoing research to determine the appropriate growth factors required for successful differentiation in nontenocyte cell lines.

In addition to cell type, cell sources in TTE literature also vary. Murine, porcine, and equine cells are often used in lieu of human tissue due to commercial availability within research settings. While these models may be cost-efficient and more readily available than human cells, the translational utility of these models is lower, given that human and animal tissues experience different types of *in vivo* mechanical stimulation. Overall, the benefits and drawbacks of each cell type and source continue to be further studied as TTE moves towards translational applications.

### 2.2. Traditional Bioreactor Systems in TTE

**Figure 3:**
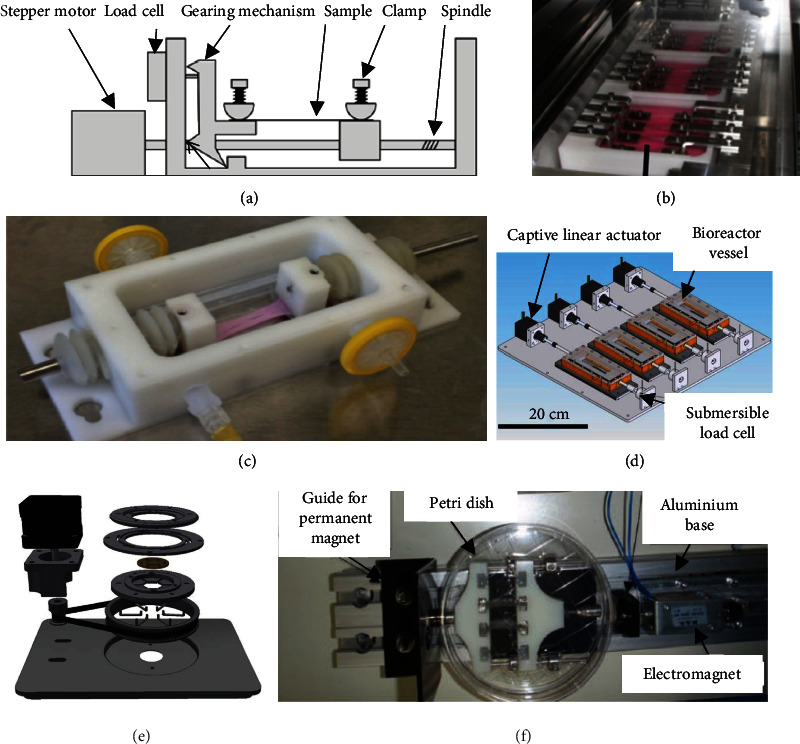
Traditional uniaxial stimulation bioreactors. (a) Cross-section of traditional stepper motor uniaxial stretch bioreactor system [74]. (b) Mechanical stimulation bioreactor for multiple tendon patches [61]. (c) Stem cell differentiation device for tendon TE [60]. (d) Scale-up of multiple parallel-running stepper motor bioreactors [78]. (e) Exploded view of tensile bioreactor presented by Schürmann et al. [79]. (f) Uniaxial stretch bioreactor system driven by electromagnet [66].

Most bioreactors being used in traditional TTE studies consist of a stepper motor actuating a tendon construct clamped to a tensile stage or contained within a tissue culture plate (Figure 3) [74]. Commercially available bioreactors using this mechanism include the ElectroForce series [37–39], Ebers TC-3 [41], and MechanoCulture T6 Mechanical Stimulation System [40, 42] for solid scaffold constructs, or the FlexCell Tissue Train [34–36] and Strex Cell ST-140-10 Mechanical Stretch Device [43] for gel based constructs on a tissue culture plate. These systems have been priced from 25,000 to upwards of 50,000 USD [75]. A number of recent studies have also developed customized tensile stage-stepper motor systems. Qin et al. presented a bioreactor which could stimulate up to four constructs simultaneously with a stepper motor and microcontroller. Cyclic uniaxial strain was applied in precise amplitudes and frequencies to their scaffolds, with rest periods in between stimulation cycles [61]. The bioreactor presented by Talo et al. combined a uniaxial tensile stage with an oscillating mechanism which provides bidirectional culture media to their tissue construct, attempting to mimic physiologic fluid flow [65]. A limited number of studies have presented tensile stage bioreactors which can provide bidirectional or different types of loading. Although Sensini et al. only applied a uniaxial load to their tissue construct, the CellScale MCB1 bioreactor used in their study can provide biaxial loading [63]. The custom-built bioreactor developed by Lee et al. relies on a group of driving motors which provide torsional stress and a second group which provide tensile stress to simulate physiologic loading experienced by the knee. The bioreactor-stimulated tissues from this study demonstrated superior mechanical properties to statically cultured constructs [67].

While several recent studies have provided alternative approaches to actuating their bioreactors, the constructs lack physiologic relevance given that these systems largely rely on uniaxial loading, involve tissue constructs that are not representative of an anatomic location for the tissue construct to be used in and offer inadequate mechanical load to tissue constructs. The diaphragm pump and pressure regulator system used in Liu et al.'s study allowed for the tissue construct to receive compressive mechanical stimulation by cyclic bulging and deformation of the membrane the tissue was cultured upon [64]. Raimondi et al. proposed a bioreactor which provided pulsatile shear stress and uniaxial tensile loading using an electromagnetic actuator. This was achieved by employing a magnet externally to a petri dish culture. The outside body of this system contains a permanent magnet and the inside body contains an electromagnet. By adjusting the current flow, a piston was electromagnetically moved back and forth and translated onto their collagen construct, resulting in the cells and the scaffold fibres aligning in the direction of loading [66]. The range of stress reported in this study, 1.5-4.5 N, is orders of magnitude lower than the load experienced by Achilles tendons *in vivo,* which can often experience loads of 6-8 times body weight (upwards of 3500 N) when running [76].

Despite progress in achieving reasonable strain ranges, the bioreactor systems presented in traditional TTE literature are fairly basic and do not faithfully replicate physiologic mechanical loading. Some researchers have suggested that uniaxial tensile loading is the only mechanical stimulation required for optimal engineered tendon growth [11, 77], but since the effects of different types and directions of strain have not been thoroughly explored in TTE literature, this suggestion has not been substantiated. Instead, based upon current evidence, it is likely that bioreactors with closer mimicry to native tendon would produce superior engineered constructs for clinical use. *In vivo* studies have consistently demonstrated that healthy tendon tissue deforms nonuniformly [23–27] and mechanobiology studies have shown that tendon cells respond uniquely to different types of loading in a directionally dependent manner [28, 29, 31]. Furthermore, mechanical testing has shown that tendon tissue will deform differently depending on anatomic location. Positional tendons, such as the supraspinatus, will deform less prior to failure, whereas energy storing tendons, such as the Achilles tendon, are more elastic than positional tendons [18, 22]. As discussed in Box 1 and Box 2, current research suggests that tendon tissue requires multiaxial loading for physiologically relevant development and that tendons have different functions and exhibit varying mechanical properties depending on their anatomic location. New bioreactor systems will be required to address the limitations of uniaxial tensile devices.

## 3. Advanced Robotics for Provision of Mechanical Loading

### 3.1. Musculoskeletal Humanoid Robots

**Figure 4:**
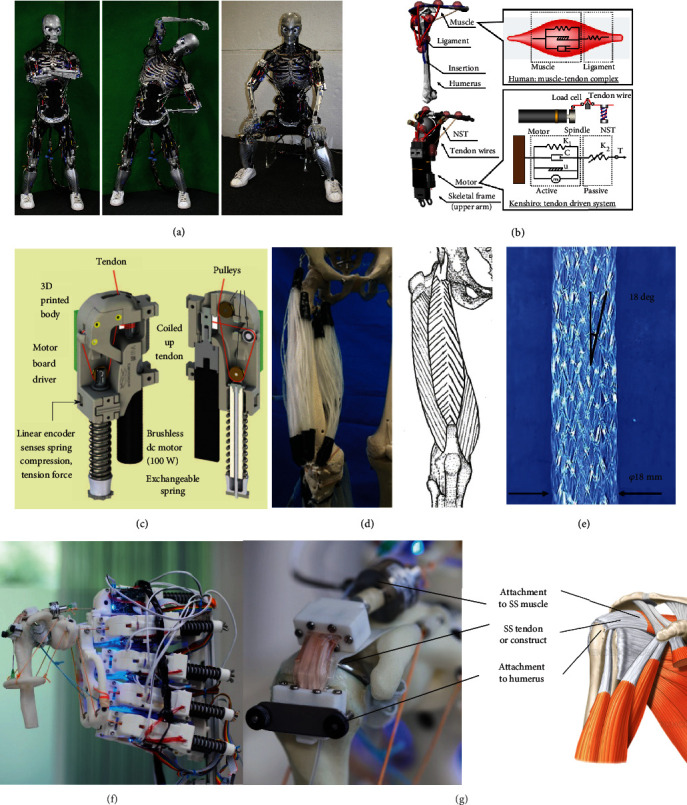
Advanced robotic technology applicable to TTE. (a) Musculoskeletal humanoid robot “Kenshiro,” built to human scale and (b) schematic showing analogy between human muscle-tendon complex and humanoid robot tendon-driven system, presented by Asano et al. [81]. (c) Schematic showing myorobotic actuator used in tendon-driven musculoskeletal robots [83]. (d) Comparison between human quadriceps muscles and multifilament musculoskeletal and (e) individual McKibben muscle filament robot presented by Kurumaya et al. [84]. (f) Adapted shoulder of musculoskeletal robot Roboy and (g) comparison between tendon tissue construct attached to Roboy's shoulder and human shoulder, presented by Mouthuy et al. [85].

To address the lack of current bioreactor systems which can provide mechanical loading in consideration of anatomic location and typical joint motion, musculoskeletal humanoid robots have been proposed as a bioreactor platform to assist in growing tissue grafts for clinical application (Figure 4) [14]. Initially designed for applications such as crash test dummies, prostheses, and athletic enhancement, there are four characteristics of human musculoskeletal anatomy and functionality that humanoid musculoskeletal robots imitate to be suitable for these applications: (i) body proportion, (ii) skeletal structure, (iii) muscle arrangement, and (iv) joint performance [80, 81]. Musculoskeletal robots currently in use include “Eccerobot,” developed by a cross-European partnership, “Kenshiro,” developed by the Inaba group in Japan, “Roboy,” developed by Devanthro GmbH, and Suzumori Endo's multifilament muscle robot [80–84].

The arrangement of actuators and joint performance of these systems are of particular interest for TTE applications. Conventional humanoid robots such as Atlas, are articulated by rigid joints that restrict movement and have fewer degrees of freedom compared to human joints, have limited energy storing capacity, and provide kinematically constrained motion. These systems use torques between rigid links or stiff joint position tracking for movement control [84]. In contrast, a number of musculoskeletal robots rely on tendon-driven myorobotic systems, which better imitate the motion and characteristics of humans compared to traditional humanoids. MSK robots such as Roboy, Kenshiro, and Eccerobot use myorobotic units consisting of a brushless dc motor which generates tension like human muscles, attachment cables which act as the tendon unit, and a motor driver board and a spring encoder which act as the neurologic system by sensing variables including tension, compression, muscle length, and temperature [80, 83]. These myorobotic units are strategically positioned based on human anatomy, with the attachment cables replicating human muscle attachment points. Kenshiro's myorobotic unit achieves flexible joint movements that are adaptive to external forces by implementing Hill's muscle model and replicates physiologically relevant behaviours, such as a tendon stretch reflex (Figure 4(b)) [81]. Both Kenshiro and Roboy use measured muscle length and tension in their musculoskeletal control system to achieve desired movements (Figure 4(c)).

In addition to the development of tendon-driven myorobotic actuators, MSK robots with multifilament muscles are an attractive alternative for TTE. The lower extremity musculoskeletal robot presented by Kurumaya et al. relies on McKibben artificial muscles (Figure 4(e)). These actuators consist of an inner bladder which expands when pressurized and a helical braided sheath which transforms the circumferential pressure into an axial contraction force. The thin multifilament McKibben muscles described by Kurumaya et al.'s study can densely connect between joints, providing muscle redundancy for completing movements, similar to the human body. Another significant advantage to the McKibben muscle is that multiple filaments can be arranged in various configurations to replicate the shapes of various human muscles, such as biceps or deltoids. The lower extremity robot presented in this study was able to reasonably replicate ankle and knee motion with muscle activation patterns that were obtained from an OpenSim lower extremity model that was developed by analysing human motion [84].

By having similar dimensions and weights to human limbs, tendon-driven and multifilament musculoskeletal humanoid robots strive to imitate the range of forces observed *in vivo* while actuating these structures [83]. Furthermore, since the number of degrees of freedom and arrangement of actuators are designed according to the anatomic position of analogous muscles, the direction and types of loading experienced by musculoskeletal robot joints can be considered physiologically relevant [83].

Mouthuy et al.'s recent pilot study demonstrates the viability of musculoskeletal humanoid robots as a platform for tissue engineering [85]. The Roboy musculoskeletal humanoid robot was implemented to imitate the motion and structure of a human shoulder. As a starting point, Mouthuy et al. focussed on low range of motion abduction-adduction movements, which mirror physiologic loading experienced by the supraspinatus rotator cuff tendon [86, 87]. A flexible tendon tissue construct with dimensions that match a supraspinatus tendon was attached to the humerus side of the humanoid robot, forming a tendon-bone interface, while the other side of the construct connected to the biomimetic tendon-driven actuators, forming a musculotendinous junction (Figure 4(g)). As an endpoint, the tissue construct cultured on the musculoskeletal robot showed increased immunofluorescence expression 14 days postculture when exposed to mechanical stimulation between 0 and 45 N loading cycles over the culture period. Although the dynamic culture on the humanoid was not found to be superior to static culture, the results of this study are encouraging as it is a first step indicating that musculoskeletal humanoids are a feasible strategy for applying mechanical stimulation. Next steps discussed in this study include working to incorporate a greater number of movements into the loading regime and completing a study with a uniaxial control to evaluate whether multiaxial loading results in a better tissue construct. With longer experiments, it would also be beneficial to explore the effect of incrementally increasing loading throughout the culture. It has been shown that incremental loading is necessary for sustained improvement in the mechanical properties of MSK tissues [64]. The capability of the humanoid robot presented in Mouthuy et al.'s study to offer multiple force loading regimes consistent with estimated quantities of force in human tendon during abduction is promising for the eventual development of physiologically relevant tissue constructs [87–90]. The cost of purchasing and modifying the Roboy arm in Mouthuy et al.'s study was around 25,000 USD.

Advantages of musculoskeletal humanoid robots compared to conventional bioreactor systems include the ability to provide multiaxial loading patterns, potential for loading in consideration of human movement patterns, and provision of loading magnitude similar to estimated *in vivo* forces. Kurumaya et al.'s lower extremity McKibben muscle humanoid has been programmed to move and recruit muscles using a model based on human kinematic data [84]. This allows for the joint structures to experience variable multiaxial loading, in contrast to conventional bioreactors which are limited to uniaxial linear tensile loading. As discussed in Box 1, tendon properties vary by anatomic location. It will therefore also be important for future bioreactors to provide anatomically targeted loading. Although one conventional study tried imitating knee loading through a combination of torsional and tensile strain [67], other conventional bioreactor systems have generally been unable to achieve this. Mouthuy et al.'s recent pilot study demonstrated the feasibility of using a musculoskeletal shoulder robot to mechanically stimulate a cell-material construct through abduction-adduction movements. The humanoid Kenshiro would also likely be able to provide loading in consideration of human movement patterns, given that the majority of its joints had similar range of motion to humans [80]. Clinically useful grafts will need to withstand loading quantities like those experienced by tendons *in vivo*. Mouthuy et al.'s supraspinatus tendon construct was exposed to tensile forces up to 45 N. This quantity is consistent predicted human supraspinatus tensile force predicted by musculoskeletal modelling and cadaveric models during abduction-adduction movement [87, 88, 90]. Conversely, the measured tensile force in a number of conventional TTE studies was found to be orders of magnitude lower than predicted *in vivo* forces [35, 48, 58, 66].

### 3.2. Soft Robotics

**Figure 5:**
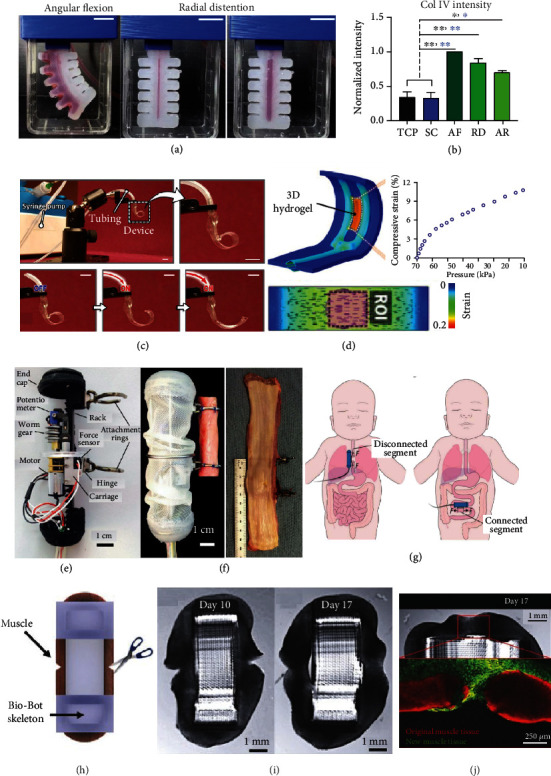
Biohybrid soft robotic actuators in tissue engineering. (a) Biohybrid soft robot, presented by Fell et al., showing changes in tissue construct with angular flexion and radial distention. (b) Increased collagen expression is seen with angular flexion (AF), radial distention (RD), or flexion and distention (AR) compared to static culture (SC) and static plate culture (TCP) [94]. (c) Paek et al.'s soft robotic constrictor in three different states of constriction. (d) Finite element model predictions of substrate geometry and strain during pneumatic actuation [95]. (e) Damian et al.'s in vivo actuation system. (f) Unrolled postop oesophagus. (g) Potential treatment locations for long-gap oesophageal atresia and short bowel syndrome from Damian et al.'s study [97]. (h) An overview of the experimental setup used by Raman et al., including induced injury to muscle indicated by arrow. (i) A macroscopic increase in muscle (black region on schema) is noted between day 10 and 17 of Raman et al.'s experiment. (j) Red stain represents old muscle tissue and new tissue was stained green on day 17 of experiment [100].

Biohybrid soft robotics is focussed on developing robots which are biomimetic and compliant (Figure 5). These systems are designed to “permit adaptive, flexible interactions with unpredictable environments” and have been proposed as a platform for medical applications such as tissue engineering [13]. Soft robotics technology can be actuated through a number of modalities, including temperature, pneumatic pressure, and light. They are made of soft materials such as hydrogels, rubber, and even engineered human skeletal muscle tissue [13, 91–93]. Although still in early development, soft robots may be particularly well-suited for actuating TTE bioreactors. Soft robotic systems offer advantages compared to conventional bioreactors given that (i) their flexible, compliant properties allow them to mimic the anatomic conformation of native tendon, (ii) they are capable of providing multiaxial actuation, and (iii) a number of the techniques used in soft robotics overlap with current TTE practices [92]. These characteristics allow for greater control in providing mechanical stimulation to tissue constructs and could allow for development of models that exhibit physiologic strain distributions [94].

Biohybrid soft robotics are already being used to mimic *in vivo* strain for smooth muscle. Fell et al. developed a biohybrid soft robot which used pneumatic actuation to achieve angular flexion up to 140° and radial distention to 20° of the tissue construct, imitating the range of motion experienced by the femoropopliteal artery during locomotion (Figure 5(a)) [94]. Tissue constructs which received multiaxial stimulation in Fell et al.'s study were shown to have greater collagen production and upregulation of smooth muscle phenotypes compared to unstimulated constructs. Paek et al. presented a soft robotic actuator which was able to induce cellular alignment and actin polymerization in mechanosensitive cell lines, including respiratory and vascular connective tissue (Figure 5(c)) [95]. Their elastomeric actuator was pneumatically regulated and capable of dynamic bending motion, mimicking the constriction of tubular organs. The system was compatible with primary culture of human endothelial cells, fibroblasts, and smooth muscle cells. The actuator was advanced as it is capable of organotypic modelling of complex tissues such as vascular networks, and the effects of compressive forces on them. Paek et al.'s actuation system could be applied to TTE to mimic stretching of a tendon that is bent or wrapped around a structure in its natural position, such as a rotator cuff tendon. Furthermore, this system is capable of coculture, an approach that has been advantageous in producing tissue constructs with morphologic similarity to tendon tissue [96].

Damian et al.'s robotic implant that provides *in vivo* tissue regeneration in a porcine model via mechanical stimulation is another approach that could be adapted to TTE [97]. The robot is designed to induce lengthening of tubular organs, such as the oesophagus and intestines, by computer-controlled application of traction forces (Figure 5(e)). The applied forces induced cell proliferation and lengthening of the organ without a reduction in diameter, while the animal was awake, mobile, and able to eat normally. Damian et al.'s system was able to provide incremental traction to the tissue construct as it developed to continuously enhance mechanical properties. This study differs greatly from the above discussed approaches as it used *in vivo* conditions and existing structures as attachment points in the subjects. This approach could be interesting if applied to two ends of a ruptured tendon, potentially in combination with a seeded graft to bridge the gap. Ideally, an implantable soft robotics system would provide incrementally decreasing support to the engineered tendon graft as seeded cells proliferate and the injured tissue heals. The success of such an intervention would depend greatly on the state of the ruptured tendon and therefore might be difficult to take into clinics. Another way this setup could be studied is *in vitro*, as an approach to form large tissue grafts for complete tendon tears. The *in vitro* setup could benefit from a whole-joint system, such as those being used for shear force studies, tribology studies on implants, and for orthopaedic intervention testing [98, 99].

Recent studies have shown that soft robots synergise well with technologies currently being explored in TTE for scaffold production. Raman et al. developed a skeletal muscle bioactuator using bioprinting capable of self-healing after being damaged (Figure 5(h)) [100]. Bioprinting is an emerging strategy in TTE which is growing in interest for establishing microarchitectural context at the scaffold level. Proposed benefits of this technique include higher initial cell densities, resulting in increased ECM production, comparable structural complexity to native tissues, and higher throughput compared to traditional scaffolding methods for eventual large-scale implementation [101–103]. Soft robotic systems can also rely on more traditional TTE technologies; a recent review has presented a number of muscle inspired coiled soft actuators have been produced using electrospinning [104].

Collectively, soft robotics technology is already being adopted in tissue engineering and has potential to help advance development of physiologically relevant tendon constructs. Longer term tissue cultures with incremental loading have not been fully explored in conventional TTE studies. Damian et al.'s *in vivo* study involving scalable loading provides vision as to how a soft robotics system may be used to fulfil this purpose. An additional benefit of these relatively small, modular systems is greater possibility of eventual *in vivo* repair of tendon injuries using robotic systems. Rigid tensile stage bioreactors used in conventional TTE studies are often limited to the development of linear rod-shaped tissue constructs which are not designed to undergo bending or shear. In contrast, native tendons wrap around bony structures and are known to undergo torsion, shear, compression, and multiaxial tensile loading simultaneously as joints move [23–27]. A significant advantage of flexible, soft robotic actuators such as those presented by Fell et al. or Paek et al. is the ability to produce anatomically relevant tissue constructs capable of undergoing multiaxial loading and bending or wrapping around other structures [94, 95]. Although soft robotic systems have not yet been implemented in TTE studies, there is good opportunity for synergy, given that these studies have already explored culturing mechanosensitive tissue, such as muscle tissue [100], and have implemented techniques which are regularly used in TTE, such as electrospinning and bioprinting.

## 4. Future Perspectives and Concluding Remarks

Recent TTE experimental work has been summarised, and limitations in current research have been discussed in this review. One of the most significant limitations in conventional TTE studies using uniaxial bioreactors has been the lack of bioreactor systems which can provide physiologically relevant mechanical stimulation. Advanced robotic technologies show promise in addressing this limitation. Musculoskeletal humanoids are developed using anthropometric data and human musculoskeletal models to have biomimetic force and movement profiles. These robots attempt to have similar dimensions to humans and their actuators are arranged to mimic the anatomic configuration of muscles [80, 84]. The ability of these systems to provide multiaxial loading in consideration of physiologic movement patterns is a significant advantage over traditional tensile stage bioreactors. To date, one study has demonstrated the viability of musculoskeletal humanoid robots for mechanically stimulating tendon constructs [85].

Soft robotic actuators are an alternative platform proposed for the advancement of current TTE research. In contrast to the hydrogel sheets or linear rod-shaped tissue constructs produced by tensile stage bioreactors, soft robotic actuators are capable of producing tissue constructs which undergo multiaxial bending movements [94]. The ability to bend or wrap around structures is an important consideration in the development of connective tissues such as tendon. We expect that soft robotics technology would synergise well with current TTE research, given that soft robotics technology has already been used to develop other connective tissue constructs, such as skeletal muscle and blood vessels [95, 100]. An end goal with TTE is translation to clinical use. Damian et al.'s recent work, which has demonstrated *in vivo* applications of soft robotics technology, helps provide vision to how this technology may eventually be used for clinical tendon repair [97].

There are a number of key challenges to consider as advanced robotic platforms are implemented in TTE. Although *in vivo* evidence suggests that different types and directions of loading are required for healthy tendon development, there is currently no literature comparing tendon constructs stimulated with traditional uniaxial loading and multiaxial physiologic loading. A main deliverable in future research would be to compare the technologies proposed in this review to current bioreactor systems available for TTE. As discussed in current TTE research, the quantity of strain experienced by tendon tissue is of critical importance to its healthy development. Traditional methods of deformation measurement such as strain gauges will be inadequate for accurately estimating multiaxial deformation. Systems capable of measuring different types of loading in 3D will be required. Digital image correlation and quantitative strain elastography are two technologies that have been explored for *in vivo* tendon imaging and may be useful in next phases of experimental work [23, 24, 50, 58]. Affordability and accessibility of advanced robotic technology will be important considerations for widespread adoption within the TTE research. Most commercially available conventional TTE bioreactor systems are priced between 25,000 and 50,000 USD, and other recent TTE studies have attempted to develop low-cost bioreactor systems using 3D printing in the range of 5,000–10,000 USD [45, 46]. In contrast, musculoskeletal humanoid robots are in their infancy and there is limited commercial availability, with the bioreactor system used by Mouthuy et al. costing 25,000 USD. Creating open access online repositories with parts lists and sharing software code through portals such as GitHub will make a development of new advanced robotic technology more affordable and accessible to researchers in this field. Regulation of soft robotic systems and musculoskeletal humanoid robots will be an important future consideration as these technologies are more widely implemented in TTE. In the United States, these would likely be classified by the Food and Drug Administration (FDA) as “Combination Biological Products” regulated by the Centre for Biologics Evaluation and Research. In European Union, engineered constructs developed using advanced robotic technology would be regulated as an “Advanced Therapy Medicinal Product” (ATMP) regulated by the European Medicines Agency.

This article has primarily discussed the importance of mechanical loading in the development of healthy tendon tissue. Considering that a number of therapeutic interventions for tendon injury involve contactless stimulation, several recent reviews have suggested that electrical and magnetic stimulation may positively contribute to tendon development and healing [7, 10]. One recent TTE study has developed a piezoelectric scaffold with potential to provide cells with electrical stimulation [58]. Working to better understand the mechanisms of these contactless techniques and evaluating their potential for use in TTE is an interesting future area of study.

In summary, tendon injuries present a tremendous socioeconomic burden and are increasing in prevalence with an ageing demographic. Tissue engineering has received considerable attention for its potential to address this growing challenge. This review article has emphasised the key role of mechanical context in producing useful tendon constructs and has highlighted musculoskeletal robots and soft robotic systems as viable strategies for advancing this field of research.

## Figures and Tables

**Figure 1:**
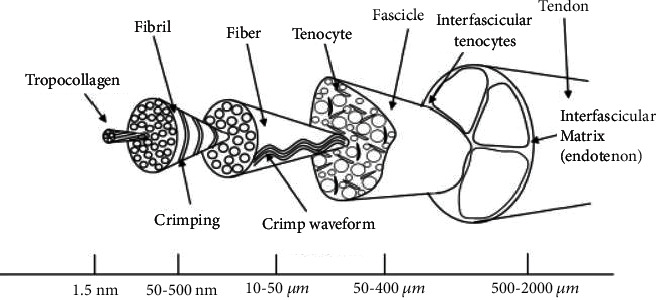
Hierarchical structure of tendon tissue (adapted from Thorpe et al.) [22].

**Figure 2:**
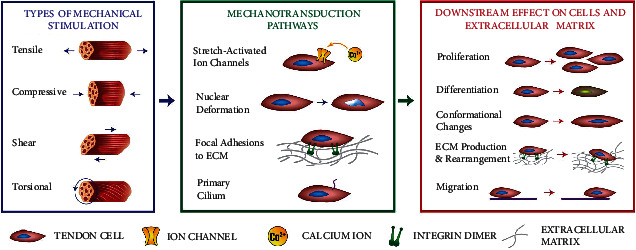
Mechanotransduction pathway in tendon tissue. Tendon tissue experiences a variety of mechanical stimuli during physiological loading (left), including tensile, compressive, shear, and torsional loading [26, 32]. These stimuli are then transduced into cellular cues (middle). The transduced signals upregulate various biochemical pathways, influencing important physiologic processes for tendon tissue such as proliferation, differentiation, homeostasis, ECM production, and conformational changes to tendon cells present within the tissue (right) [1, 32].

**Box 1:**
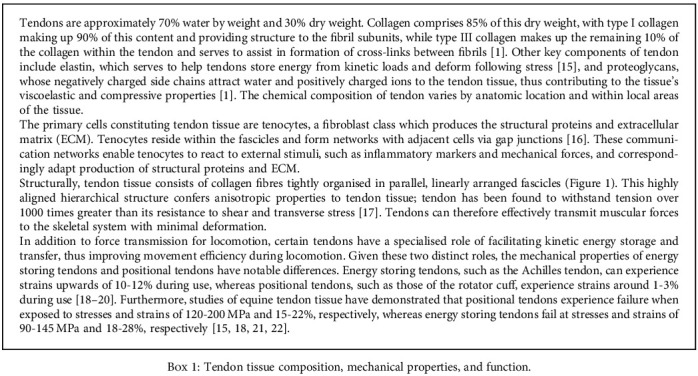
Tendon tissue composition, mechanical properties, and function.

**Box 2:**
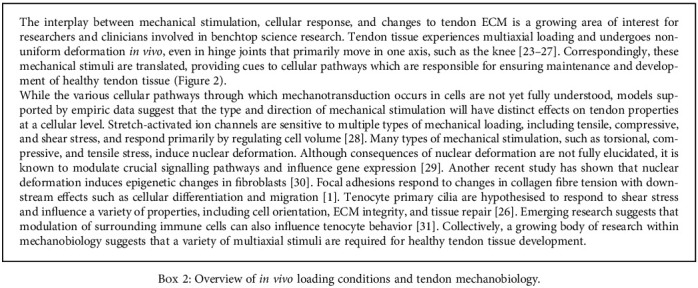
Overview of *in vivo* loading conditions and tendon mechanobiology.

**Table 1:** Recent TTE experiments involving provision of mechanical stimuli through traditional robotic platforms.

Bioreactor system	Types and directions of mechanical stimulation	Reference	Loading frequency and duration	Measured load	Measured strain	Strain distribution	Cell source and tissue construct details (dimensions)
Flexcell® tissue train culture system	Uniaxial tensile	Wee et al. [34]	1 h/day over 1-14-day period at 0.5 Hz	Not available	0-5%	Modelled as uniform	Tonsil-derived MSCs seeded to Dulbecco's modified eagle medium in UniFlex bioreactor plates
		Ciardulli et al. [35]	4 h/day over 11-day period at 1 Hz	9 × 10^−2^ pa	10%	Modelled as nonuniform	hBMSCs seeded in a hyaluronate/polylactic-co-glycolic acid hydrogel (30 mm × 20 mm × 4.5 mm)
		Mubyana and Corr [36]	8 h loading cycles over 7-day period at 0.1 Hz	0-100 kN/m^2^	0-0.70%	Modelled as uniform	HDF seeded in agarose growth channels

Bose ElectroForce® bioreactor	Uniaxial tensile	Baumgartner et al. [37]	3 h/day over 7-day period at 1 Hz	Not available	10%	Modelled as uniform	Human adipose-derived stem cells (ADSCs) seeded to electrospun poly(vinylidene fluoride-co-hexafluoropropylene)/polydimethylsiloxane (PDMS) patches (1 cm × 1 cm × 200 *μ*m)
		Chen et al. [38]	3 h/day over 7-day period at 1 Hz	Not available	6%	Modelled as uniform	Leporine tenocytes seeded to electrospun hyaluronic acid-(PCL)/platelet rich plasma fibre scaffolds (length: 5 mm; width: 5 mm)
		Yuan et al. [39]	2 h/day over 7-day period at 0.25 Hz	Not available	3%	Modelled as uniform	hBMSCs seeded to electrospun poly(L-lactic acid) (PLLA) scaffold coated with type I collagen and chondroitin sulphate (50 mm × 10 mm × 120 *μ*m)
		Garcia Garcia et al. [40]	Twice daily for 1 h over 5-12-day period at 1 Hz	0-250 kN/m^2^	5%	Modelled as uniform	Rat BMSCs seeded to electrospun (PCL) mats (40 mm × 12.5 mm × 120 *μ*m)

Ebers TC-3 bioreactor system	Uniaxial tensile	Deniz et al. [41]	30 min/d for 2 days then 1 h/day over 10-day period at 0.33 Hz	Not available	3-6%	Modelled as uniform	Human tenocytes seeded to polyglycerol sebacate sheet (surface area: 1 cm × 2.5 cm)

MCT6 bioreactor	Uniaxial tensile	Wu et al. [42]	2 h/day over 12-day culture period at 0.5 Hz	Not available	4%	Modelled as uniform	hADSCs seeded to electrospun polycaprolactone (PCL) scaffold (length: 1.2 cm; width: 1.2 cm; thickness: 180 *μ*m)

Strex STB-140-10 stretching system	Uniaxial tensile	Nam et al. [43]	Continuous over 6-72 h period at 0.5-1 Hz	Not available	4-12%	Modelled as uniform	hBMSCs seeded to silicon chambers (surface area: 10 cm^2^)
	Uniaxial tensile	Chen et al. [44]	8 h/day over 6-day period at 0.25 Hz	Not available	6%	Modelled as uniform	Murine tendon derived stem cells (TDSCs) seeded to CelGro culture scaffold (25 mm × 5 mm)
	Uniaxial tensile	Janvier et al. [45]	5 h/day for 5 days/week over 3-week period at 0.5 Hz	Not available	5%	Modelled as uniform	hBMSCs seeded to fibrin hydrogel in 3D-printed PLLA wells (7 mm × 8 mm × 4 mm between construct attachment points)
	Uniaxial tensile	Banik and Brown [46]	2 h/day over 21-day period at 0.01 Hz	Not available	3-6%	Modelled as uniform	hMSCs seeded to electrospun type I collagen and PCL encased in a 3D-printed frame (length: 2.8 cm; diameter: 6.4 mm)
	Uniaxial tensile	Atkinson et al. [47]	8 h/day over 14-day period at 0.67 Hz	0.1-25 kN/m^2^	10%	Modelled as nonuniform	Equine tenocytes suspended in 200 *μ*l collagen solution

Custom uniaxial tensile stage bioreactors	Uniaxial tensile	Sawadkar et al. [48]	Dynamic trial: 15 min then 15 min rest, repeated over 24 h period; static trial: constant loading over 24 h period	0-3700 *μ*N (dynamic) 3250 *μ*N (static)	0-10% (dynamic) 10% (static)	Modelled as uniform	Rabbit tibial tendon fibroblasts seeded to collagen lattice derived from rat tail (75 mm × 25 mm × 15 mm)
	Uniaxial tensile	Hsiao et al. [49]	Continuous 8 h period at 0.5 Hz	Not available	4 or 8%	Modelled as nonuniform	Rat patella tenocytes, cultured in wells on a tensile plate (no scaffold)
	Uniaxial tensile	Morita et al. [50]	Continuous over 2-day period at 1 Hz	Not available	2-8%	Modelled as nonuniform	hBMSCs seeded to polydimethylsiloxane coated glass membrane (20 mm × 20 mm × 10 mm)
	Uniaxial tensile	Nakanishi et al. [51]	Static loading over 4-8-week period	Not available	Not available	Modelled as uniform	HDFs seeded into 3D-printed DMEM spheroids (diameter: 500 *μ*m–600 *μ*m)
	Uniaxial tensile	Grier et al. [52]	40 min/day over 6-day period at 1 Hz	Not available	10%	Modelled as nonuniform	hBMSCs seeded to bovine collagen-glycosaminoglycan (GAG) scaffold (length: 15 mm)
	Uniaxial tensile	Raveling et al. [53]	Continuous over 3-day period at 0.05 Hz	0-190 kN/m^2^	0-10%	Modelled as uniform	Murine MSC cells seeded to bovine type I collagen sponges (length: 12 mm; width: 4 mm)
	Uniaxial tensile	Engebretson et al. [54]	0.5-2 h/day for culture of up to 7 days at 0.01-0.03 Hz	Not available	2%	Modelled as uniform	Rat BMSCs seeded to decellularised human umbilical vein (diameter: 6.75 mm; length: 6.5 cm)
	Uniaxial tensile	Testa et al. [55]	1 h/day over 15-day period at 0.5 Hz	Not available	10%	Modelled as uniform	Murine embryonic fibroblast cells seeded to crosslinked polyethylene glycol (PEG)-fibrinogen hydrogel (10 mm × 3.3 mm × 1.5 mm)
	Uniaxial tensile	Grier et al. [56]	10 min every 6 h over 6-day period at 1 Hz	Not available	10%	Modelled as uniform	hBMSCs seeded to collagen-GAG scaffold extracted from bovine tendon (6 mm × 6 mm × 15 mm)
	Uniaxial tensile	Joo Kim et al. [57]	Continuous 12 h period at 0.5 Hz	Not available	2-4%	Modelled as nonuniform	Rat tail tenocytes suspended in DMEM and tested in moulded polydimethylsiloxane bioreactor wells (26 mm × 19 mm × 1 mm)
	Uniaxial tensile	Cook et al. [58]	1 h/day over 5-day period at 0.5 Hz	0-5 mN	1-9%	Modelled as nonuniform	hADSCs seeded in electrospun fibrin/alginate scaffold bundles (1.5 cm × 3.0 cm × 1.0 cm)
	Uniaxial tensile	Burk et al. [59]	15-30-60 min loading alternated by 15-30-60 min rest at 1 Hz	Not available	2%	Modelled as uniform	Equine ADSCs seeded to decellularised equine tendon scaffold (10 mm × 1 mm × 2 mm)
	Uniaxial tensile	Youngstrom et al. [60]	0.5-1 h/day over 8 days at 0.33 Hz	Not available	0-5%	Modelled as uniform	Equine BMSCs seeded to decellularised equine tendon scaffolds (10 mm × 45 mm × 400 *μ*m)
	Uniaxial tensile	Qin et al. [61]	20 min/h for 12 h for 7-day period at 0.2 Hz	Not available	3%	Modelled as uniform	Canine BMSCs seeded to decellularised canine tendon (length: 40 mm; width: 300 *μ*m)
	Uniaxial tensile	Cardwell et al. [62]	30 min over 3-day period at 0.25 Hz	Static tensile loading of 50 mN (daily)	4%	Modelled as uniform	Murine fibroblasts seeded to electrospun polyurethane mesh (length: 4.5 cm; width: 1 cm)

MCB1 bioreactor	Biaxial tensile	Sensini et al. [63]	1 h/day on day 3 and 6 over 7-day period at 1 Hz	Not available	5%	Modelled as uniform	Foreskin HDFs seeded to electrospun PLLA/collagen (cross-section: 1.46 ± 0.08 mm; length: 89.4 ± 2.1 mm)

Custom diaphragm pump bioreactor	Uniaxial compressive	Liu et al. [64]	3-9 h/day over 14-day period at 0.8 Hz	Not available	5-16%	Modelled as nonuniform	hBMSCs seeded in PEG-norbornene hydrogel discs (ø–9 mm, h–250 *μ*m)

Custom oscillating uniaxial tensile stage bioreactor	Uniaxial tensile	Talo et al. [65]	15-30-60 min loading alternated by 15-30-60 min rest 2 times/day over 7-day period at 0.33 Hz	4-25 N	1-7%	Modelled as uniform	Rabbit BMSCs seeded to decellularised equine tendon tissue (43 mm × 5 mm × 3 mm)

Custom perfused uniaxial tensile stage bioreactor	Uniaxial tensile, shear stress	Raimondi et al. [66]	30 min alternating cyclic and static loading over 7-14-day culture period at 0.5-2 Hz	1.5-4.5 N	3-10%	Modelled as uniform	Porcine Achilles tenocytes, seeded to equine type I collagen sponge scaffold (40 *mm* × 5 *mm* × 5 *mm*)

Custom torsional and uniaxial tensile bioreactor	Uniaxial tensile, torsional	Lee et al. [67]	Cyclic loading for 1-7-day periods at 1 Hz	Not available	10% tensile; 45°-90° torsion	Modelled as uniform	hBMSCs, seeded to decellularised porcine tibialis tendon (length: 12 cm; width: 1 cm)

## Data Availability

The data used to support the findings of this review article are available from the corresponding author upon request.
